# Tyrosine Hydroxylase, Vesicular Monoamine Transporter and Dopamine Transporter mRNA Expression in Nigrostriatal Tissue of Rats with Pedunculopontine Neurotoxic Lesion

**DOI:** 10.3390/bs8020020

**Published:** 2018-02-01

**Authors:** Lisette Blanco-Lezcano, Esteban Alberti-Amador, Mei-Li Díaz-Hung, María Elena González-Fraguela, Bárbara Estupiñán-Díaz, Teresa Serrano-Sánchez, Liliana Francis-Turner, Javier Jiménez-Martín, Yamilé Vega-Hurtado, Isabel Fernández-Jiménez

**Affiliations:** 1 Experimental Neurophysiology Department, International Center of Neurological Restoration (CIREN) Ave. 25 No. 15805 e/158 and 160, Playa, Havana 11300, Cuba; alberti@neuro.ciren.cu (E.A.-A.); mldiaz@neuro.ciren.cu (M.-L.D.-H.); marie@neuro.ciren.cu (M.E.G.-F.); baby@neuro.ciren.cu (B.E.-D.); teresa@neuro.ciren.cu (T.S.-S.); yvega@neuro.ciren.cu (Y.V.-H.); ifernandez@neuro.ciren.cu (I.F.-J.); 2 Latinoamerican School of Medicine, Km 3½ Carretera Panamericana, Santa Fé. Playa, Havana 19148, Cuba; 3 Experimental Group: “Experimental Models for Zoo-Human Sciences”, Faculty of Sciences, Tolima University, 42nd Street, Barrio Santa Elena, Parte Alta, CP 730001, Colombia; lilycolcuba@gmail.com; 4 Department of Physiology, Otago School of Medical Sciences, University of Otago, P.O. Box 913, Dunedin 9016, New Zealand; javierneuro@gmail.com

**Keywords:** pedunculopontine nucleus, substantia nigra pars compacta, tyrosyne hydroxylase, vesicular monoamine transporter 2, dopamine transporter, dopaminergic homeostasis

## Abstract

Background: The degeneration of the pedunculopontine nucleus (PPN) precedes the degeneration of the nigral cells in the pre-symptomatic stages of Parkinson’s disease (PD). Although the literature recognizes that a lesion of the PPN increases the vulnerability of dopaminergic cells, it is unknown if this risk is associated with the loss of capability of handling the dopaminergic function. Methods: In this paper, the effects of a unilateral neurotoxic lesion of the PPN in tyrosine hydroxylase (TH), vesicular monoamine transporter 2 (VMAT2) and dopamine transporter (DAT) mRNA expression in nigrostriatal tissue were evaluated. Three experimental groups were organized: non-treated rats, NMDA-lesioned rats and Sham-operated rats. Results: Seven days after the PPN lesion, in nigral tissue, TH mRNA expression was higher in comparison with control groups (*p* < 0.05); in contrast, VMAT2 mRNA expression showed a significant decrease (*p* < 0.01). DAT mRNA expression showed a significant decrease (*p* < 0.001) in the striatal tissue. Comparing nigral neuronal density of injured and control rats revealed no significant difference seven days post-PPN injury. Conclusions: Findings suggest that the PPN lesion modifies the mRNA expression of the proteins associated with dopaminergic homeostasis at nigrostriatal level. It could represent vulnerability signals for nigral dopaminergic cells and further increase the risk of degeneration of these cells.

## 1. Introduction

The degeneration of the dopaminergic neurons in the substantia nigra pars compacta (SNpc) leading to the onset of typical motor symptoms of Parkinson’s disease (PD) is preceded by a pre-symptomatic stage characterized by olfactory and sleep disorders among other pre-motor signs [1]. In this context, the early degeneration of the pedunculopontine nucleus (PPN), located in the ventrolateral part of the caudal mesencephalic tegmentum, has been studied [2,3,4,5]. The PPN sends a cholinergic and glutamatergic projection to the SNpc [6,7,8]. Post-mortem studies performed on the brains of Parkinsonian patients have shown that cholinergic pontine neurons are widely damaged [9,10,11].

Previous studies have pointed out that considerable PPN lesions induce a Parkinsonian syndrome in monkeys [12,13,14]. Excitotoxic lesion of the PPN was documented to induce nigral neuronal death in rats [15,16], while unilateral PPN lesion induced oxidative stress events in both the SNpc and striatum [17,18].

These findings have suggested that pontine-nigral projection might contribute to the survival of dopaminergic neurons in the adult brain and this effect might progressively disappear in PD [19]. The literature describes a close reciprocal morphofunctional relationship between nigral dopaminergic neurons and cholinergic pontine neurons, which makes them mutually dependent on one another to maintain survival and homeostasis [20].

The survival of nigral neurons is closely related to the maintenance of nigrostriatal dopaminergic homeostasis [21]. This process is associated with the capability of these cells to synthesize, store and release dopamine (DA) [21]. Dopamine is synthesized from the amino acid tyrosine by the action of the enzymes tyrosine hydroxylase (TH) and decarboxylase, of the aromatic amino acids [22,23]. Following this, DA is stored in synaptic vesicles after uptake by the vesicular monoamine transporter 2 (VMAT2) [24,25]. Dopamine stored in vesicles is released by exocytosis to the synaptic cleft [22,26]. Extracellular DA is recaptured at the pre-synaptic terminal by the action of a dopamine transporter (DAT). Once this has occurred, DA can be stored in vesicles again by VMAT2 or degraded by monoamine oxidase B (MAO-B) to form hydrogen peroxide (H_2_O_2_) and 3,4-dihydroxyphenylaldehyde [22,26].

However, the effect of the neurotoxic injury of the PPN on the expression/activity of proteins involved with the maintenance of dopaminergic homeostasis is still unknown. Tyrosine hydroxylase catalyzes the rate-limiting step in the biosynthesis of dopamine [22,27]. Dopamine is a highly reactive molecule with a high tendency to form reactive oxygen species (ROS) and neurotoxic quinones, through its metabolism in the cytosol [22,28]. Thus, the vesicular packaging of DA by VMAT2 prevents the oxidation processes and therefore the neurotoxic effects of endogenous dopamine [25,29,30]. On the other hand, a DAT function to rapidly take up DA from the extracellular space into the presynaptic neuron is essential for regulating the magnitude and duration of dopaminergic signaling [31].

Neurotoxic injury of the PPN causes redox changes in nigral tissue [17]. The literature recognizes that the smallest peroxide exposure of neurons induces adaptive responses including the expression of antioxidant proteins as well as proteins and enzymes that guarantee the vitality of neuronal metabolism and biosynthesis machinery [32]. However, this initial effect can be lost if the presence of ROS is significantly increased [33]. In this case, we hypothesized that the oxidative stress environment present in the nigral tissue very soon after neurotoxic injury of the PPN may contribute to modifying the expression of proteins that determine dopaminergic homeostasis. These changes in the early stage of PD may have long-term consequences that deepen the degenerative course of the nigral dopaminergic neurons. Thus, the purpose of this work was to investigate the effects of a PPN lesion on nigral TH, VMAT2 and striatal DAT mRNA expression in rats.

## 2. Materials and Methods

### 2.1. Experimental Subjects

Male, Wistar rats weighing 200–350 g, from the Centre for the Production of Laboratory Animals (CENPALAB), Mayabeque, Cuba, were housed 5 per cage under a temperature of 22–24 °C, with a relative humidity of 60 ± 5% and a light–darkness cycle of 12–12 h. Water and food were provided ad libitum. Experiments were carried out in accordance with the Cuban Regulations for the Use of Laboratory Animals (CENPALAB 1997) and were approved by the Ethical Committee of the International Center for Neurological Restoration.

### 2.2. Surgical Procedure

The surgical procedure was carried out according to that published [17,18]. Briefly: the rats received an anesthesia injection [chloral hydrate (420 mg/kg weight), intraperitoneal (i.p.)]. A stereotactic frame for rodent surgery (David Kopf Instruments, Tujunga, CA, USA) was utilized to perform the neurotoxin injection. The right PPN was selected for the neurotoxin administration. Stereotactic coordinates and data on the concentration and volume of neurotoxin administered are shown in Table 1. Also, a brief description of the experimental procedure is shown in Table 1. Sham lesion of the PPN: the surgical procedure was the same as that followed for injection of the neurotoxin. In place of the neurotoxin the rats received 0.5 μL of physiological saline solution.

### 2.3. Molecular Biology Studies

Sample collection: the animals were sacrificed by decapitation, after being anesthetized with a lethal dose of chloral hydrate (480 mg/kg i.p.). The procedure for obtaining the nigral and striatal tissue have been previously described [17,18]. Taking into account that, in rats, the pontine–nigral projections are fundamentally ipsilateral [35], the molecular studies were performed on the right nigrostriatal tissue, ipsilateral to the PPN lesion. Molecular biology studies were performed in 5 rats of each experimental group.

RT-PCR analysis for gene expression of dopaminergic proteins: TH, VMAT2 and DAT.

Dopaminergic protein mRNA expression was studied at 48 h and 7 days after the PPN neurotoxic lesion. The total RNA was extracted from the nigral and striatal tissue of 25 rats using TRIzol^®^ Reagent (Invitrogen). The first-strand cDNAs were synthesized using the Go Taq G2, Hot Star Reverse Transcription System (Promega) (www.promega.com/Go Taq G2 USA) in accordance with the protocol manufacturer’s instructions. Table 2 shows the data concerning the sequence of each primer used for RT-PCR, the annealing temperature (according to the size of the primers previously designed) and the size of each gene product. Cycling conditions were the recommended according protocol manufacturer’s instructions: one cycle a 45 °C for 45 min; 40 cycles for 2 min and 30 seg at 94 °C and extension temperature at 68 °C for 7 min).

PCR products were separated by 2% agarose/ethidium bromide gel electrophoresis and visualized under UV light. Each electrophoresis was performed twice, followed by a semi-quantification analysis. For the semi-quantitative analysis, the free online program ImageJ (Version 1.44; Wayne Rasband, National Institute of Health, Bethesda, MD, USA; http://imagej.nih.gov/ij) was used. The analysis was performed according to the published method [36,37]. The background activity of the target band was subtracted and then normalized using β-actin as a reference.

### 2.4. Morphological Studies

The morphological studies were performed in 5 rats of each experimental group).

Rats were anesthetized with chloral hydrate (420 mg/kg body weight, i.p.) and perfused transcardially with phosphate-buffered saline (PBS) followed by 4% formaldehyde. The brains were removed and blocked in the coronal plane and paraffin-processed.

Six sets of 21 coronal sections (10 µm of thickness) were taken from the anterior cerebellum through to anterior substantia nigra and placed on gelatin-coated slides. Every section was heated, deparaffinized and rehydrated. Two series corresponding to PPN coordinates were stained according to Cressyl Violet protocol in order to locate the correct site of neurotoxin injection.

### 2.5. Immunohistochemistry

Immunohistochemical studies were performed on these paraffin-embedded sections using the avidin-biotin-complex method. One series composing of twelve coronal sections of SN (the coronal sections that swept the SN approximately from the coordinate AP = −5.16 mm to the coordinate AP = −6.48 mm were considered) was stained with antibody against tyrosine hydroxylase (TH) (Rabbit polyclonal antibody, 1:500; Santa Cruz Biotechnology, Inc., Dallas, TX, USA, TH (H-196): sc-14007).

The neuron count was performed only in the sections corresponding to right SN from NMDA-lesioned rats and Sham-operated rats groups. The images were obtained by means of a high-resolution digital camera (DP 71) coupled to a conventional optical microscope (Olympus BX 51, Tokyo, Japan). Once the images were captured, the cell count was performed. Cell numbers were counted following a systematically random sampling scheme. Neuronal characteristics included the presence of fibers; a clearly defined nucleus with a pale and a dark, condensed nucleolus. The counting was performed manually using the cell counter plugin of the free online program ImageJ (Version 1.44; Wayne Rasband, National Institute of Health; http://imagej.nih.gov/ij). Neuronal density (number of cells/mm^2^) was estimated by dividing the number of cells between the areas of a complete field of the 40× objective (0.137 mm^2^). This work was done by researchers blind to the experimental group being studied.

### 2.6. Data Processing and Statistical Analysis

The values were expressed as mean ± SEM. Normal distribution and homogeneity of variance of the data were analyzed applying the Kolmogorov-Smirnov and Levene tests, respectively. The comparison between experimental groups of the TH, VMAT2 and DAT mRNA expression was carried out by a one-way analysis of variance (ANOVA) followed by a multiple range test of Duncan. The comparison between NMDA-lesioned rats and Sham-operated rats of the neuronal density was carried out by Mann-Whitney U Test. For all analyses, a significance level of 0.05 was considered using the Statistica 8.0 software (StatSoft Ink, Tulsa, OK, USA) software.

## 3. Results

### 3.1. Morphological Studies

**Figure 1:**
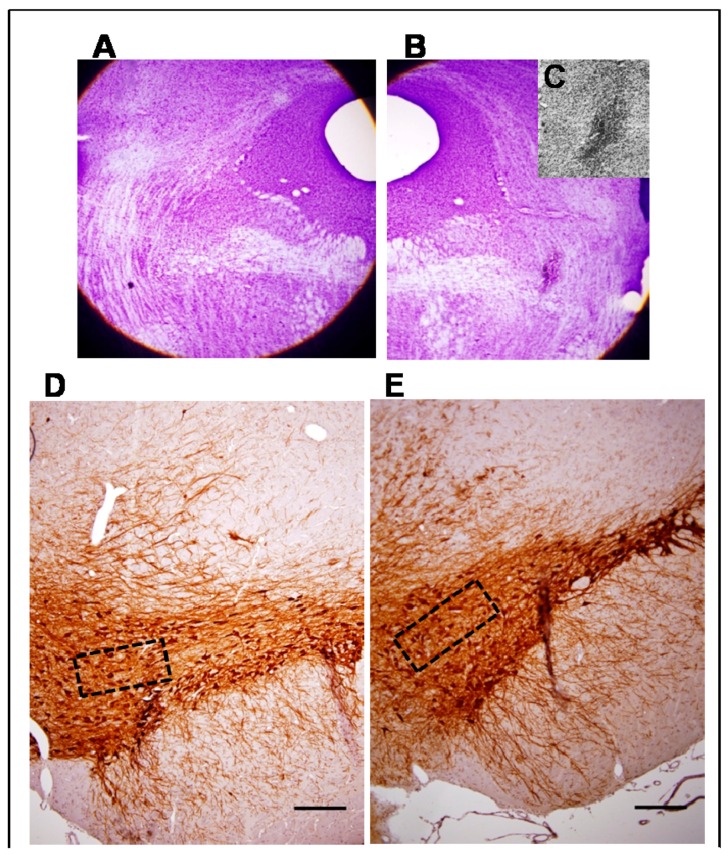

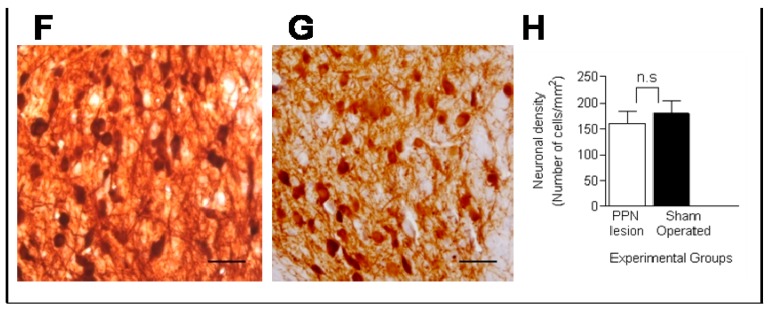
Morphological study. (**A**,**B**) Composition made from photomicrographs of brain coronal sections of a seven-day lesioned rat. (**A**) PPN area from the left side, contralateral to the lesion; (**B**) Cresyl Violet study revealed the zone of injury in the area occupied by the right PPN adjacent to the superior cerebellar peduncle (4×); (**C**) Magnification of the lesion area (10×); (**D**–**G**) Representative photomicrographs of the immunohistochemical study for tyrosine hydroxylase in nigral coronal sections of: Sham-operated (**D**) and NMDA-lesioned rats (**E**) (4×). The area within the rectangle of each image is magnified (40×) in (**F**) and (**G**) respectively. Note that dopaminergic cell bodies are well conserved in the SNpc on both sides; (**H**) Comparison of neuronal density showed non-significant differences between the right SNpc from NMDA-lesioned rats and Sham-operated rats groups (*n* = 5 for each experimental groups). (Scale bar for D and E = 100 µm; scale bar for F and G = 50 µm).

Morphological evaluation showed that the stereotactic injection of NMDA solution covered the full extent of the anterior–posterior plane of the PPN (approximately between the stereotactic coordinates AP −7.20 mm to AP −8.16 mm). Cresyl Violet study revealed the zone of injury in the distal part of the decussation of the superior cerebellar peduncle (Figure 1A–C). The lesion does not appear to compromise this bundle of fibers.

The immunohistochemical study for TH revealed the integrity of the SNpc ipsilateral to the PPN lesion (Figure 1D–G). No dopaminergic neural depopulation area was observed in the coronal sections of the right SNpc. Dopaminergic neurons were healthy with well-preserved cell bodies and projections. No cell debris suggestive of ongoing cell death processes was observed (Figure 1D–G). Comparison of neuronal density showed non-significant differences between the right SN from NMDA-lesioned rats and Sham-operated rats groups (Z = 1.84 *p* > 0.05) (Figure 1H).

### 3.2. Molecular Biology Studies

TH and VMAT2 mRNA expression was studied in nigral tissue while DAT mRNA expression was investigated in striatal tissue.

**Figure 2:**
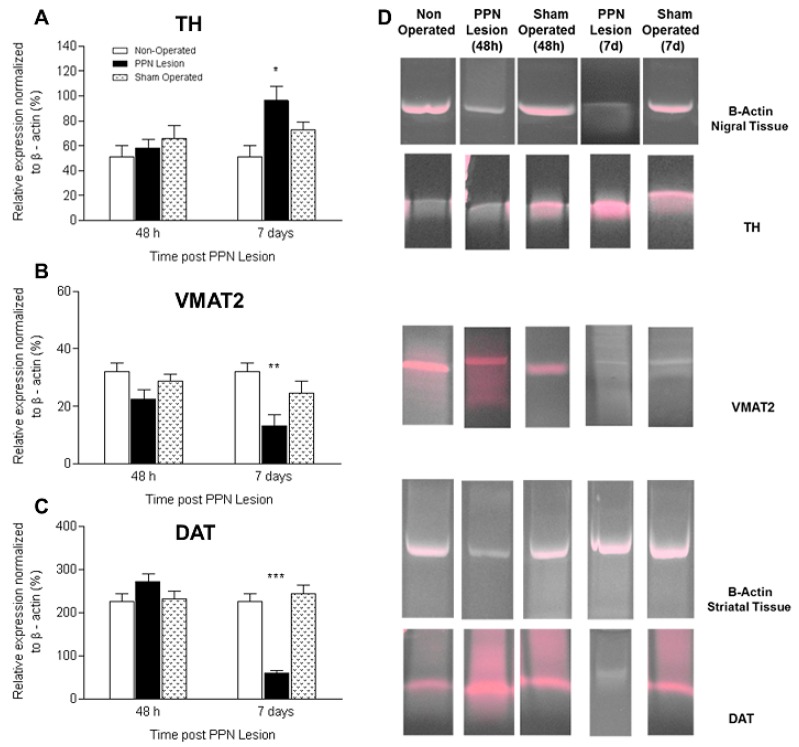
Effect of pedunculopontine nucleus (PPN) neurotoxic lesion on the mRNA expression of proteins related to dopaminergic homeostasis in nigral and striatal tissue. The study was by semi-quantitative RT-PCR, expressed as percentage of b-actin 48 h and 7 days after PPN lesion. Note that for the three proteins studied there were no significant differences between experimental groups 48 h after PPN injury (*p* > 0.05). (**A**) Comparison between experimental groups of tyrosine hydroxilase mRNA expression in nigral tissue 7 days after PPN lesion (F_(2, 12)_ = 5.34 *p* < 0.05); (**B**) Comparison between experimental groups of vesicular monoamine transporter 2 (VMAT2) mRNA expression in nigral tissue 7 days after PPN lesion (F_(2, 12)_ = 6.16 *p* < 0.01); (**C**) Comparison between experimental groups of dopamine transporter (DAT) mRNA expression in striatal tissue 7 days after PPN lesion (F_(2, 12)_ = 20.29 *p* < 0.001); (**D**) Agarose gel electrophoresis bands representative of the semiquantitative RT-PCR study for each of the markers studied. Experimental groups: non-operated (*n* = 5), PPN lesion (*n* = 5), and sham-operated rats (*n* = 5). * *p* < 0.05; ** *p* < 0.01; *** *p* < 0.001. Bars represent mean ± SEM.

Significant differences were not appreciated in the mRNA expression of TH, VMAT2 and DAT 48 h after PPN lesion, in none of the experimental groups (*p* > 0.05) (Figure 2A–C).

Seven days after PPN lesion, the levels of TH mRNA expression in nigral tissue of lesioned rats was significantly higher than control groups (F_(2,12)_ = 5.34 *p* < 0.05) (Figure 2A).

Regarding VMAT2 mRNA expression, there was a trend for a decrease in nigral tissue 48 h after the PPN lesion; however, significance was not achieved. Seven days after the pontine lesion, the decrease VMAT2 mRNA expression reached significance between the injured rat groups (F_(2,12)_ = 6.16 *p* < 0.01) (Figure 2B).

In connection with the DAT mRNA expression this variable showed a significantly decrease in striatal tissue (F_(2,12)_ = 20.29 *p* < 0.001), seven days after NMDA injection (Figure 2C).

## 4. Discussion

The major findings of this work demonstrate that a neurotoxic lesion of the PPN induces early changes in the protein mRNA expression related to nigrostriatal dopaminergic homeostasis in the rat brain. These changes can be interpreted differently depending on the protein analyzed. The increase and the decrease of the TH and DAT mRNA expression, respectively, could be considered a plastic change in response to the cellular stress associated with the compromise of the pontine-nigral projection. However, the decrease found in the VMAT2 mRNA expression could be interpreted as a sign of very early damage in nigral dopaminergic neurons. It is very interesting that these functional changes precede the morphological changes as no morphological changes were observed in the SNpc seven days post-PPN lesion.

The literature recognizes that DA neurons require maintenance of a certain range of activity, which guarantees their physiological functioning and their own survival [19]. This is consistent with the neuroprotective role that has been attributed to the pontine-nigral projection through which the cholinergic excitatory input reaches the dopaminergic neurons [38,39]. On the other hand, the role of dopamine as an endogenous neurotoxin has been discussed [26,40]. When the level of cytosolic DA outside the synaptic vesicles is increased, the processes of oxidation (mediated by MAO-B) and auto oxidations are stimulated, forming cytotoxic ROS and quinones [41,42,43,44].

Our group has demonstrated that 48 h after the PPN lesion, changes occur in the cellular and molecular nigral scenario, characterized by a transient increase in the gluthatione (GSH) content and the brain-derived neurotrophic factor (BDNF) mRNA expression [18]. GSH protects cells against exogenous and endogenous toxins including ROS [45]. On the other hand, BDNF is involved in the survival of nigral DAergic neurons [46]. It is possible that the very early plastic changes observed by us played a positive role in maintaining unaltered dopamine biosynthesis machinery. This fact could explain that no significant differences were found between NMDA-lesioned rats and control groups in the VMAT2, DAT and TH mRNA expression in the nigrostriatal tissue 48 h post-PPN injury.

At the same time, our previous studies confirmed a slight increase in catalase activity and malondyaldehyde (MDA) concentrations from 48 h and most notably seven days after PPN injury in nigral and striatal tissues [17].

It is well known that a rupture in the balance between ROS production and the ability of antioxidant defense systems to detoxify intermediate metabolites leads to protein damage and dysregulation in the cellular metabolism [47]. Different transcription factors are regulated by the ROS-induced modifications that lead downstream to changes in the expression of different gene families [33,48].

In relation to the gene expression of TH, the complexity of its regulation could respond to the importance of the functional activity of this enzyme [27,49,50]. An AP-1 sequence (the Fos/Jun immediate early protein-binding site) has been identified ~200 bp upstream of the TH transcription start site [27]. AP-1 is a general term used to denote a family of transcription factors that control the expression of many genes by binding to their promoter sites [48]. The interesting aspect in this case is that AP-1 is known to be induced by H_2_O_2_ [50]. ROS are responsible for inducing the expression of many genes, modulating transcription factors such as AP-1 [48,51,52,53,54].

The literature indicates that modifications in the TH mRNA expression may respond to intracellular redox status [22]. It is possible that the nigral oxidative stress environment associated with PPN neurotoxic injury is playing a key role in the increase of TH mRNA, which may be related to the positive regulation of ROS on AP-1. However, future studies will be necessary to confirm this hypothesis.

Higher TH mRNA levels could stimulate dopamine synthesis. However in this situation, the decrease in VMAT2 mRNA represents a major problem for the nigral dopaminergic cell. The significant decrease in VMAT2 mRNA expression in the SNpc one week after PPN neurotoxic injury may lead to alterations in dopamine vesicular storage. This signal may be potentially toxic to the dopaminergic cell [55].

Under physiological conditions, most of the intracellular dopamine is stored in synaptic vesicles [25]. Vesicular packing avoids the accumulation of DA and its subsequent oxidation to potentially neurotoxic molecules [25]. The level of VMAT2 expression is very important to proper dopaminergic functioning [56]. Mice with decreased dopamine vesicular storage capacity showed lower values of striatal dopamine along with high cysteinyl adducts of dopamine metabolites concentrations [42].

The essential role of dopamine vesicular storage and the deleterious impact when this process is interrupted have been published [29].

The nigrostriatal oxidative stress environment may also affect the expression and function of DAT [57,58]. DAT in rat brain contains cysteinyl residues that can be oxidized by ROS or quinones with subsequent modifications to the expression and function of the protein [58,59].

Decreased DAT mRNA may lead to increased levels of extracellular DA. Higher levels of extracellular DA may contribute to maintain the vitality of the nigrostriatal synapse and thereby dopaminergic control over the striatum. However, taking into account that the extracellular antioxidant activity is lower than the intracellular antioxidant activity, the increase of the extracellular DA can consequently cause the accumulation of extracellular ROS [57,60].

On the other hand, other authors have pointed out a significant decrease in the TH cell number in the SNpc after PPN lesion [20]. These results do not contradict our morphological findings. In the above-mentioned work, the morphological study was performed seven weeks after injection into the PPN of Dtx-UII, a neurotoxin selective for cholinergic pontine neurons. Our morphological studies on the SNpc were performed seven days after NMDA injection. We should consider two aspects: the difference in neurotoxins employed and the post-injury time evaluated in each study. It is possible that seven days as post-injury time the morphological changes that could be seen in a longer time window are not yet evident.

The literature indicates that dopaminergic mishandling can lead to the degeneration of nigral dopaminergic neurons with different intensity degree and progression [25]. DAT and VMAT2 are essential for normal DA neurotransmission [61]. Mice that express ≈5% of normal VMAT2 (VMAT2 LO) showed DAT protein levels and functions reduced [42]. Positron emission tomography (PET) imaging studies of Parkinsonian patients have shown that there is already lower level of DAT density in the pre-motor stage [62]. This finding has been interpreted as a compensatory mechanism in the early stages of the disease, when dopaminergic function is still relatively preserved [62]. The combination of lower DAT mRNA expression in striatal tissue and slight decrease in VMAT2 mRNA expression in nigral tissue may be a strong sign of nigrostriatal vulnerability.

Most of the experimental models of PD developed in rodents are based on the administration of selective toxins to dopaminergic neurons, such as 1-methyl 4-phenyl 1,2,3,6–tetrahydropyridine (MPTP) and 6-hydroxydopamine (6-OHDA) [63,64,65,66]. These neurotoxins directly damage the soma of dopaminergic neurons or their striatal projections [63]. From the knowledge that PPN degeneration precedes nigral degeneration, the interest in developing selective or complete lesions of the PPN that mimic the pre-symptomatic stages of PD has grown exponentially [17,18,20,67,68]. The most important strength of these approaches to the pre-symptomatic models lies in the fact that the SNpc is not directly damaged. Instead, nigral vulnerability is increased to the events that converge in neurodegeneration through the compromise of pontine-nigral projection.

Seven days post-injury represents an early temporal window that may not fully reflect the magnitude of the risk of dopaminergic neurons. Aspects that seven days after the injury were defined as a plastic change (the behavior of TH and DAT mRNA expression) or as an early sign of damage (VMAT2 mRNA expression), perhaps a little longer after the injury (30 days or more) could be considered to be a compensatory change and an irreversible damage respectively. Therefore, it would be very interesting, in future works, to study the effect of PPN neurotoxic injury on the proteins expression related to dopaminergic homeostasis in a longer time window.

On the other hand, to deepen the effect of the pontine lesion on the nigrostriatal dopaminergic homeostasis, it would be useful to study the expression of transcription factors (such as Pitx3 and Nurr 1) in whose signaling cascades are located the genes that code for TH, VMAT and DAT [69]. At the same time, the study of the concentrations of TH, VMAT and DAT proteins in the nigrostriatal tissue would provide more elements to elucidate the effect of the PPN lesion on dopaminergic homeostasis.

The authors would like to add that the RT-PCR studies are semiquantitative and therefore have limitations that are avoided in quantitative PCR studies. However, in our work, the standardized steps in the literature were followed, which minimized these limitations. Initially, B-actin was studied in all samples, which was a guarantee that the RNA was correctly isolated. Subsequently the study of each marker was executed separately. The bands corresponding to each sample were processed together with the background of each of them. Finally, the band of each sample was divided by the value of the band of B-actin corresponding to that sample. This last step homogenized any possible difference between gels for the background and at the same time it was possible to compare the optical densities between groups. In each gel, a positive and a negative control were provided as a quality control. However, for future molecular studies, the representative image of the complete gel corresponding to the electrophoretic run of each marker studied could be shown.

## 5. Conclusions

Neurotoxic lesion of the PPN modifies nigrostriatal redox balance. It is possible that the oxidative stress environment favors alterations in mRNA expression of several proteins responsible for nigrostriatal dopaminergic homeostasis. Changes in dopaminergic homeostasis may increase nigral vulnerability to events, leading to neurodegeneration. It is difficult to extrapolate these results to the clinic. However, if the early degeneration of the PPN induces dopaminergic mishandling, this may be a key factor to consider in the treatment strategies of Parkinsonian patients in pre-symptomatic stages of the disease.

## Figures and Tables

**Table 1:** Important aspects of the procedure followed to injure the right pedunculopontine nucleus of the rats. The injection coordinates of the neurotoxin were determined taking the Bregma point as the reference [34].

Neurotoxin employed	N-methyl-D-aspartate (NMDA) (Sigma, St. Louis, MO, USA) Concentration: 0.1 M, Volume: 0.5 µL Rate: 0.1 μL/min by means of 1-μL Hamilton syringe
Stereotactic coordinates (mm)	AP: −7.80, ML: 1.60, DV: 7.60
Experimental design	In order to study the early effect of the PPN neurotoxic lesion on the gene expression of TH, VMAT2 and DAT, the mRNA expression of the above-mentioned proteins was evaluated 48 h and 7 days after injury. The following experimental groups were organized: Healthy rats (non-operated) (*n* = 12), rats-PPN injury (48 h post lesion) (*n* = 13), rats-PPN injury (7 days post lesion) (*n* = 12), rats Sham operated (48 h after surgery) (*n* = 13), rats Sham operated (7 days after surgery) (*n* = 12). Rats were lesioned, assigned randomly to the experimental groups and sacrificed in the temporal window already described

**Table 2:** Sequence of primers for RT-PCR.

Gene Product	Sequence of Primers	Product Length (bp)	Annealing Temperature (°C)
TH	5′ TTC CCC ATG TTC AAC GGA CC-3′ 5′ GCG AGC ACA GTA ATC ACC TTC-3′	449	56
DAT	5′ GGA AGC TGG TCA GCC CCT GCT T-3′ 5′ GAA TTG GCG CAC CTC CCC TCT G-3′	266	60
VMAT2	5′ CGC AAA CTG ATC CTG TTC AT-3′ 5′ AGA AGA TGC TTT CGC AGG TG-3′	175	60
B ACTIN (endogenous control)	5′ ATT TGG CAC CAC ACT TTC TAC A-3′ 5′ TCA CGC ACG ATT TCC CTC TCA G-3′	379	55
